# Slice-level diffusion encoding for motion and distortion correction^☆^

**DOI:** 10.1016/j.media.2018.06.008

**Published:** 2018-08

**Authors:** Jana Hutter, Daan J Christiaens, Torben Schneider, Lucilio Cordero-Grande, Paddy J Slator, Maria Deprez, Anthony N Price, J-Donald Tournier, Mary Rutherford, Joseph V Hajnal

**Affiliations:** a Centre for the developing Brain, King’s College London, London, UK; b Philips, Guildford, UK; c Centre for Medical Image Computing and Department of Computer Science, University College London, London, UK

**Keywords:** MRI, Diffusion, Motion correction, Microstructure, Foetal imaging, EPI, TA:, acquisition time, AP:, anterior-posterior, SNR:, Signal to Noise Ratio, ADC:, Apparent diffusion coefficient, ROI:, Region of Interest, ssEPI:, single-shot EPI, SAO:, slice acquisition order

## Abstract

Advances in microstructural modelling are leading to growing requirements on diffusion MRI acquisitions, namely sensitivity to smaller structures and better resolution of the geometric orientations. The resulting acquisitions contain highly attenuated images that present particular challenges when there is motion and geometric distortion. This study proposes to address these challenges by breaking with the conventional one-volume-one-encoding paradigm employed in conventional diffusion imaging using single-shot Echo Planar Imaging. By enabling free choice of the diffusion encoding on the slice level, a higher temporal sampling of slices with low b-value can be achieved. These allow more robust motion correction, and in combination with a second reversed phase-encoded echo, also dynamic distortion correction. These proposed advances are validated on phantom and adult experiments and employed in a study of eight foetal subjects. Equivalence in obtained diffusion quantities with the conventional method is demonstrated as well as benefits in distortion and motion correction. The resulting capability can be combined with any acquisition parameters including multiband imaging and allows application to diffusion MRI studies in general.

## Highlights

• Breaks the conventional one-volume one-encoding paradigm in diffusion MRI.
• Higher temporal sampling of anatomically reliable b0 slices.
• Allows more robust distortion and motion correction.
• Additional benefits include quicker scans by reduced thermal heating.

## Graphical abstract

**Figure:**
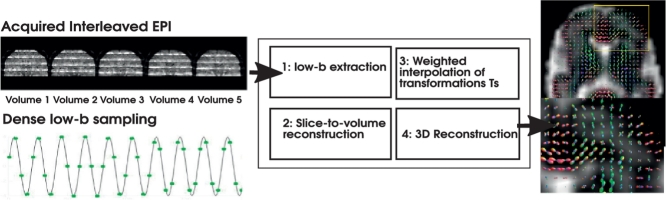

## 1 Introduction

### 1.1 Increasing requirements on diffusion data

Diffusion MRI (dMRI) offers a unique observation window into tissue microstructure in-vivo (Le Bihan et al., 1986). Increasingly advanced biophysical modelling techniques for dMRI allow insight into microscopic tissue properties, such as axon diameter (Assaf, Blumenfeld-Katzir, Yovel, Basser, 2008, Alexander, Hubbard, Hall, Moore, Ptito, Parker, Dyrby, 2010), neurite morphology (Zhang et al., 2012), global connectivity patterns (Tournier, Calamante, Connelly, 2012, Wedeen, Wang, Schmahmann, Benner, Tseng, Dai, Pandya, Hagmann, D’Arceuil, de Crespigny, 2008, Steven, Zhuo, Melhem, 2014) and cell size and density (Panagiotaki et al., 2015). These techniques demand a rich dMRI acquisition for accurate parameter estimation, namely a high number of samples varying in direction, described by the unit vector *b*-vector and strength, expressed in *b*-value in the diffusion encoding space. Typically, a set of different orientations acquired on one *b*-value are referred to as one shell.

Recently, novel techniques to change the diffusion encoding itself have emerged, including oscillating gradients (Baron and Beaulieu, 2014) and *b*-tensor encoding strategies (Szczepankiewicz, Lasič, van Westen, Sundgren, Englund, Westin, Ståhlberg, Lätt, Topgaard, Nilsson, 2015, Topgaard, 2017). A common factor across these dMRI acquisition techniques is the need for higher *b*-values – containing important information for accurate parameter estimation in biophysical modelling techniques. But the corresponding higher diffusion encoding strength, achieved by stronger attenuation with higher amplitude gradients, poses additional challenges:

• High-*b* data is typically low in anatomical contrast and Signal-to-Noise Ratio (SNR). This hampers important post-processing steps such as correction for geometric distortion and for artifacts due to motion during the acquisition. Both often rely on registration approaches.
• The required strong gradients pose significant demands on the hardware regarding thermal heating (duty cycle) and power supply, resulting in longer acquisition times to allow for cooling periods.

DMRI data is most often acquired using Spin-Echo single-shot Echo-Planar Imaging (ssEPI) (Turner et al., 1991). One common artifact in EPI is geometric image distortion due to magnetic field susceptibility. In addition, while EPI techniques are quick enough to freeze intra-slice motion, they are intrinsically planar techniques and do not resolve inter-slice motion. Therefore, the acquired stacks of slices needed to capture whole volumes typically feature inconsistent and variable slice locations. Especially rotation between slice acquisitions break the intra-volume consistency and renders interpolation between slices almost impossible. Recent accelerations with multiband imaging, a technique acquiring multiple slices at the same time and using geometrical coil information for subsequent separation (Barth et al., 2016) has the benefit of locking multiple slices together. However, novel challenges regarding for example the slice acquisition order and cross-talk artefacts (Hutter et al., 2018) arise.

Inconsistencies originating from motion and distortion, are, however, a major impediment for accurate estimation of biophysical parameters. Variations due to inconsistent slice locations perturb these measurements and hamper accurate estimation of any biophysical parameter. Therefore, a vast body of work has been proposed to deal with motion and distortion artifacts and to produce truly 3-dimensional accurate representations of the object.

Regarding distortion correction, traditional methods often either acquire additional data to model the distortion - by mapping the polarising magnetic field (B0) directly, by obtaining separately acquired reversed phase encoding volumes to estimate the field (Jezzard and Balaban, 1995) or by using information in undistorted anatomical images to provide an estimate for the B0-field through image registration strategies.

However, most previous approaches share the limitation to obtain a static B0-field estimation, relying on data acquired before or after the scan and thus unable to estimate the dynamic effects of distortion. Furthermore, all these techniques except the direct mapping of the B0-field, require a registration step in post-processing, hampered for high-*b* data by the attenuation and thus limited anatomical contrast. The only methods to overcome these shortfalls are approaches relying on phase estimation from subsequent volumes or echos (Hutton, Bork, Josephs, Deichmann, Ashburner, Turner, 2002, Cordero-Grande, Price, Ferrazzi, Hutter, Christiaens, Hughes, Hajnal, 2018).

Methods for static motion correction can be split into real-time approaches, attempting to measure the motion within the acquisition (Aksoy, Forman, Straka, Skare, Holdsworth, Hornegger, Bammer, 2011, Kober, Gruetter, Krueger, 2012) or to acquire breath-hold data (Kim et al., 2008), and post-processing techniques attempting to estimate the motion state from the data itself.

Extensive research has been dedicated to reconstructing 3D volumes from scattered slices in anatomical imaging using slice-to-volume (SVR) techniques (Rousseau, Glenn, Iordanova, Rodriguez-Carranza, Vigneron, Barkovich, Studholme, 2006, Kuklisova-Murgasova, Quaghebeur, Rutherford, Hajnal, Schnabel, 2012, Kainz, Alansary, Malamateniou, Keraudren, Rutherford, Hajnal, Rueckert, 2015, Gholipour, Estroff, Warfield, 2010, Tourbier, Bresson, Hagmann, Thiran, Meuli, Cuadra, 2015). Recent improvements include machine-learning approaches to predict the motion-state and parameters (Hou et al., 2017).

When extended to dMRI, all these approaches share the challenge of the strong signal attenuation and the absence of consistent anatomical features at high-*b*, which makes these images poorly suited for standard image registration (Ben-Amitay et al., 2012). They can be classified according to their proposed treatment of high-*b* data:

(a) Approaches attempting to register high-*b* data to a reference low-*b* volume typically employ mutual information to accommodate the contrast differences (Maes, Collignon, Vandermeulen, Marchal, Suetens, 1997, Oubel, Koob, Studholme, Dietemann, Rousseau, 2012, Jiang, Xue, Counsell, Anjari, Allsop, Rutherford, Rueckert, Hajnal). But they have been shown to lack in accuracy in regions such as the cortical rim (Ben-Amitay, Jones, Assaf, 2012, Nilsson, Szczepankiewicz, van Westen, Hansson, 2015, Rohde, Barnett, Basser, Marenco, Pierpaoli, 2004).
(b) Methods relying on simulating the contrast properties of high-*b* data from the low-*b* data as target for the registration were proposed based on CHARMED (Ben-Amitay et al., 2012) CSF-corrected models (Nilsson et al., 2015), Gaussian processes (Andersson and Sotiropoulos, 2015) or diffusion encoding (Scherrer et al., 2012).
(c) Approaches extrapolating the motion parameters obtained from conventional registration of the low-*b* data to the high-*b* value data based on spatial or temporal proximity. This was demonstrated with 2D liver imaging (Mazaheri et al., 2012). Recent work combines (b) and (c) by employing average targets per shell as an input for sequential slice registration (Kurugol et al., 2017) or includes multiband imaging (Marami et al., 2016).

These approaches rely in some way on the anatomically reliable low-*b* data points to estimate motion parameters throughout the acquisition.

The spacing of these low-*b* points is therefore of key importance but has, to date, not been part of active method development. All available studies are based on conventional ssEPI acquisitions. These are, however, restricted in the available sampling freedom due to the employed *volume view* rather than *planar view*. In conventional dMRI all slices in a volume are acquired with a given diffusion weighting before repeating all slices with the next chosen encoding. This results in a suboptimal non-uniform spacing in time of low-*b* data points.

### 1.2 Foetal imaging as the application of choice

All the described challenges, motion and distortion artifacts, are major obstacles for all dMRI acquisitions. While typically less motion is observed in adult brain scans, applications such as the acquisition of data from abdominal organs tend to suffer from increased motion. The presented methods are general and can be applied to any such application. However, in the following we demonstrate the specific challenges and successful application of our technique on in-utero imaging of the foetus.

In-utero imaging is prone to motion artifacts due to maternal breathing and the foetal movement itself. Furthermore, changes of the maternal pose due to respiration, as well as the proximity of gas in the maternal bowel can result in *time-varying* susceptibility induced distortions. Traditional techniques that assume static single time point field maps are unhelpful in this scenario, and the move from 1.5T to 3T for advanced foetal studies has exacerbated these problems, particularly with long scan durations needed for eloquent dMRI data.

### 1.3 Overview

In this study we propose a fundamental change on the acquisition side to answer the aforementioned challenges and problems. We hypothesize that these will contribute to more eloquent data, suitable for more accurate motion and dynamic distortion correction.

We break with the traditional paradigm of *one volume, one diffusion encoding* (described in Section 2). Our method offers enhanced flexibility to choose the diffusion encoding per slice rather than per volume, and thus allows the sampling of low-*b* slices with a higher and more-uniform density (Section 2.1–2.3). In addition, acquisition of a second spin-echo with reversed phase encoding at each ssEPI shot (Section 2.4) provides data usable for distortion correction for each individual slice (Gallichan et al., 2010). The combination of these elements ensures that there can be low-*b* information suitable for distortion and motion correction obtained at high temporal resolution. Candidate post-processing strategies designed to exploit this new data structure are presented in Section 2.2 and Section 2.5. Finally, experiments on phantoms and in-vivo on adult and foetal subjects, as well as simulations are presented (Section 3). Their results (Section 4) depict the ability of the proposed acquisition and processing methods to improve motion and distortion correction. Possible extensions and limitations are discussed in Section 5.

These concepts were introduced in Hutter et al. (2017), but have been significantly extended and tested in this paper. We present a more general formulation of the proposed diffusion acquisition approach, and simulations to illustrate its performance and limitations. We also present new motion correction results, and new phantom and adult scans.

## 2 Materials and methods

**Fig. 1:**
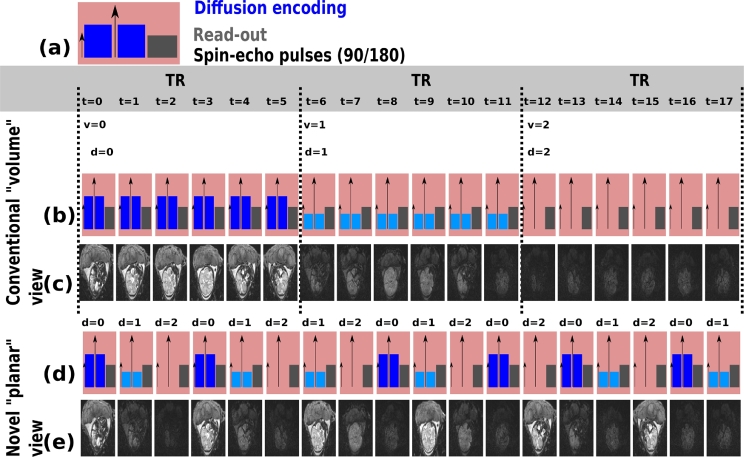
A schematic illustration of the acquisition scheme for one EPI slice is depicted in (a), consisting of the diffusion encoding in blue, the pulses required for the spin echo in black and the read-out train in gray. (b-c) depict a conventional volume-wise acquisition and (d-e) the proposed slice-wise acquisition. The chosen parameters are $N_{s}=6,$$N_{d}=3$. Thereby, (b) and (d) are schematics and (c) and (e) depict exemplary axial foetal brain images. (For interpretation of the references to colour in this figure legend, the reader is referred to the web version of this article.)

ssEPI is an intrinsically *planar* acquisition - each slice is acquired independently with an EPI sequence block, consisting of the diffusion preparation (blue in Fig. 1a) and the EPI read-out (grey in Fig. 1a). A full dMRI EPI acquisition with a chosen number of diffusion encodings, $d=1,.,N_{d},$ and a fixed number of slices $s=1,.,N_{s},$ consists in total of $N_{t}=$ *N_d_* · *N_s_* slices. Every slice can be labelled by its temporal index *t* with $t=1,.,N_{t},$ within the sequence.

The acquisition is split in equal blocks or volumes *v* with $v=1,.,N_{d}$ containing *N_s_* subsequent slice excitations - each acquiring all slices specified within one geometric volume. The geometric location *z*, is defined by the slice acquisition order (SAO). SAO relates the index *s* to the geometric location *z* by z=SAO(s). Examples are ascending SAO, resulting in SAO(s)=s or the frequently used odd-even SAO ([1,3,5,7,9,.,2,4,6,8,.]). The optimal choice allows sufficient recovery of the longitudinal magnetization after the excitation and acquisition of one slice before locations in spatial proximity are excited. Higher degrees of motion warrant more distant subsequent excitations. The same SAO is preserved over all volumes to achieve a regular excitation pattern.

### 2.1 Slice parameterization

Each slice is thus parameterized by its geometric location *z*, its order within the acquisition *t* and the index of the volume *v* it is part of. In addition, each slice acquisition varies by its diffusion preparation – specified by the chosen diffusion encoding *d*. This contains for Stejskal–Tanner encoding the diffusion strength (*b*-value) and diffusion sensitization direction (*b*-vector).

In conventional dMRI, the choice of *d* is linked intrinsically to the volume *v* by the *one volume - one encoding paradigm:* Within volume v=1, all *N_s_* slices are acquired with encoding d=1, as illustrated in Fig. 1b–c. *N_s_* repetitions of the same sequence block lead to a full volume and define the repetition time (TR) of the sequence. In a next volume (v=2), diffusion encoding d=2 is selected and all slices $s=1,.,N_{s}$ are acquired. This process is repeated until all encodings *N_d_* are acquired.

The novel proposed slice-level diffusion encoding breaks with this rigid relation between volume and encoding. Instead, it allows the intrinsic flexibility of the planar acquisition to be exploited and considers all slices to be independent regarding their diffusion encoding.

Each slice is parameterized by the global slice index *t* as well as *v, s* and *z*. Thereby, the volume index *v* and the volume slice index *s* are calculated from the global index *t* by division by the number of slices: *v* equals the result rounded to the next integer and *s* equals the remainder after division. The geometric slice index *z* is directly obtained from the slice acquisition order *SAO*:

(1) $$\begin{array}{ccc} & & \left(t,d,v,s,z\right)\;\text{with}\;v=\left\lfloor t/N_{s}\right\rfloor +1,\\ & & \;\;s=t\;\text{mod}\;N_{s}+1\;\text{and}\;z=SAO\left(s\right). \end{array}$$

As illustrated in Fig. 1d, the diffusion encoding now changes from slice to slice, leading to subsequent slices having free diffusion encoding (Fig. 1e).

### 2.2 Superblock & interleaving

Individual slice encoding allows very great flexibility and can be optimised on a variety of time and length scales, including treating the whole acquisition as a single non-repeating sequence of differently weighted slices. However, for the above mentioned goal to facilitate motion and distortion correction it is helpful to closely interleave low and high *b*-values. It can also be advantageous to ensures that complete volumes of encoded data are achieved even if the scan is interrupted or abandoned. Therefore, we present in the following a **Superblock & Interleaving** approach.

**Fig. 2:**
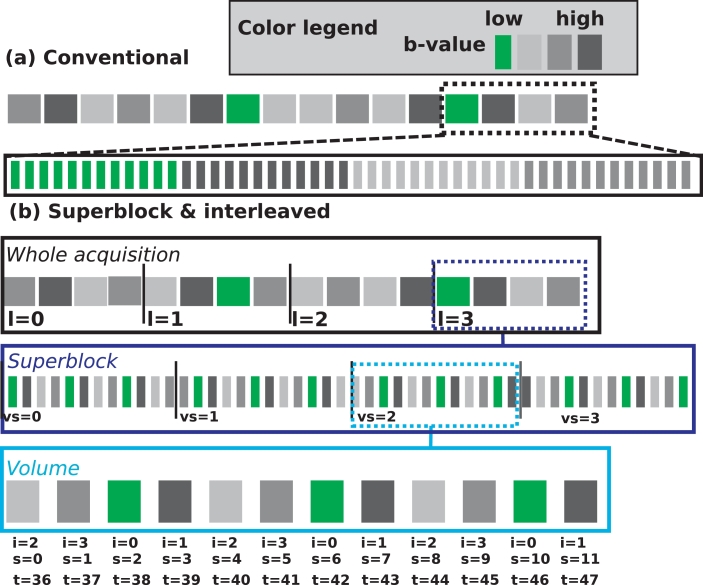
The structure of the conventional EPI sequence (in (a)) and the proposed Superblock & Interleaving (in (b)) are depicted. In (a) the whole acquisition is shown together with a zoom into the last four volumes in the second row. In (b), a whole acquisition ($N_{s}=12,N_{d}=16,L=4$ is shown in the first row. The second row shows a zoom into the superblock l=3. Finally, in the third row, volume vs=2 within this superblock is shown together with all relevant parameters (*i, s, t*).

**S**uperblock The sequence of slices is sub-divided into so called superblocks with length *L*. Each superblock consists of *L* · *N_s_* slices (or *L* volumes) with *L* chosen diffusion encodings. Thereby, *L* consecutive diffusion samplings were intertwined so that all slices are acquired with all diffusion weightings (i.e. *b*-value shells) after *L* volumes. The number of required blocks depends on the total number of diffusion samples: $N_{l}=N_{d}/L$.
**I**nterleaving Within each superblock, however, every volume interleaves all *L* diffusion encodings. These are laid out sequential in time and shift from one volume to the next by the shift factor *f*—chosen in the simplest case as 0. This process is formulated in Algorithm 1 and graphically depicted in Fig. 2b.

**Algorithm 1:**
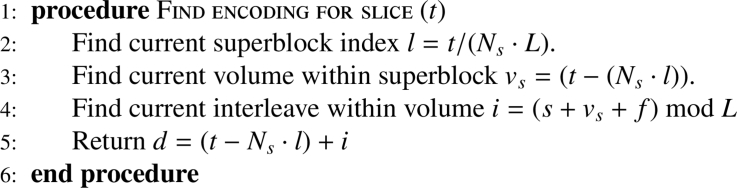
Calculate encoding per slice.

The interleave & superblock approach reduces to the conventional approach for L=1. It enforces one additional constraint: the superblock length *L* needs to be a divisor of the number of slices: $N_{s}\;\text{mod}\;L=0$.

### 2.3 Motion estimation—higher temporal low-*b* sampling

The optimal acquisition sequence depends on the nature of the considered motion. The major source of motion in most applications, breathing motion, is characterized by its smooth and quasi-regular pattern in time. Assuming a physiological breathing rate of 10–12 inhale-exhale cycles per minute, and thus a nominal periodicity of around 5 s. Respiration induced displacements tend to be smooth rather than jerky and dominantly in the anterior-posterior (AP) and superior-inferior directions.

**Fig. 3:**
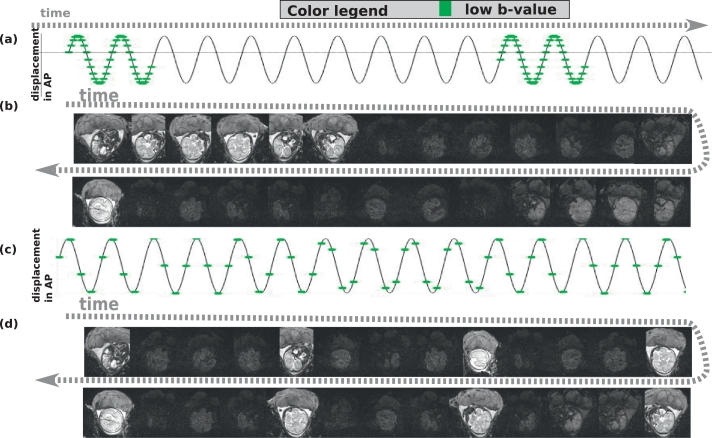
The distribution of the low-*b* slices is illustrated schematically for conventional (a-b) and superblock and interleaved (c-d) acquisition. Thereby, the displacement in anterior-posterior direction originating from a (simplified) exemplary breathing pattern is illustrated schematically in (a) and (c) in black. In green, the temporal indices of low-*b* slices are indicated. In (b) and (d) slices obtained at sub-sampled temporal locations along (a) and (c) are schematically depicted to illustrate the temporal sampling density of low-*b* slices. (For interpretation of the references to colour in this figure legend, the reader is referred to the web version of this article.)

Fig. 3a visualizes for illustrative purposes the displacements in AP direction of such a typical breathing cycle in parallel to the acquired EPI slices plotted in (undersampled) temporal order of acquisition using a conventional volume-based sequence. With typical TRs between 6 and 12 sec in foetal imaging, each acquired volume thus experiences the displacement of 1–3 breathing cycles. This corresponds, assuming a single-slice acquisition time of around 200 ms, to roughly 25 slices.

Fig. 3b also illustrates the varying *b*-values and thus resulting anatomical contrast over the depicted volumes. It indicates the low contrast and decreased SNR for higher *b*-values. This translates into a considerable variation in the reliability of the estimated transformations if using slice-to-volume registrations. The temporal index of low-*b* slices is marked on the respiratory plot in green - indicating the dense sampling of the respiration in the first volume but globally unbalanced sampling density.

To improve sampling—specifically to increase the sampling density of low-*b* throughout the acquisition, the proposed interleaving diffusion sampling is employed as follows: Within a superblock with length *L*, both low-*b* and high-*b* encodings are included (there are L−1 high *b*-values). The subsequent interleaving leads to a uniform spread of the low-*b* slices over time, as illustrated in Fig. 3c and d for a superblock structure with L=4 encodings, here illustrated with one b=0, one b=400 and two $b=1000\frac{s}{{\text{mm}}^{2}}$ volumes. The exact parameterization and choice of the ratio of low-*b* to high-*b* depends on the expected motion pattern, the repeat time (TR) and ratio of low- vs. high-*b* in the planned diffusion encoding scheme.

So far, these concepts were discussed and visualized only as a function of the temporal slice index *t* (or *s* within the volume) but without considering the geometric location. But to ensure optimal registration properties, the low-*b* value data was spread out not only maximally in time to densely sample motion patterns but also in space to ensure spatial proximity of every high-*b* slice to a low-*b* slice.

**Fig. 4:**
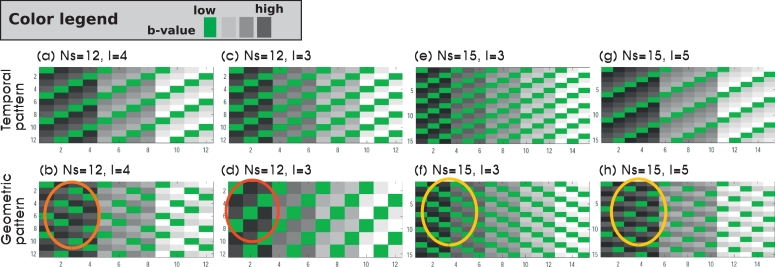
Illustrations of the temporal (first row, a,c,e,g) and spatial (second row, b,d,f,h) patterns of low-*b* (green) and high-*b* (gray) slices are given. The same odd-even slice acquisition order [0,2,4,6.1,3,5,7.] is used for all. The varied parameters include the number of slices *N_s_*: 12 in (a–d) and 15 in (e–h) and the superblock length *L*: 4 in (a–b), 3 in (c–d), 3 in (e–f) and 5 in (g–h). The coloured circles mark a suboptimal (red), non-uniform (orange) and to uniformly spread and optimal patterns (yellow). (For interpretation of the references to colour in this figure legend, the reader is referred to the web version of this article.)

In the following, a geometrical optimal sampling will thus be characterized by resulting in equal geometrical distance between all acquired slices with the same encoding *d* per volume *v*, corresponding to equal inter-slice inter-shot distances. Fig. 4 illustrates the relation between temporal and geometrical order for odd-even SAO for four settings of *N_s_* and *L*. Thereby, Fig. 4(a and b) illustrate the setting as given in Fig. 2 with $N_{s}=12$ and L=4, resulting in a non-uniformly spread distribution in *z*-direction (b), whereas Fig. 4(c–d) with $N_{s}=12$ and L=3 show a very suboptimal spatial result with two subsequent slices sampled back-to-back. (e-h) illustrate an example with $N_{s}=15,$ resulting in two different but equally nicely spread spatial patterns for L=3 and L=5 in (f) and (h). As a general rule of thumb, an optimal pattern can, for given even-odd slice acquisition order, only be achieved with an odd number of slices *N_s_* as shown in more detail in the Appendix.

### 2.4 Double echo for dynamic distortion correction

A common approach is to acquire the whole acquisition in one phase encoding direction, for example AP, and then at the beginning or end an additional b=0 volume with reversed phase encoding direction. This data is used to estimate a field map, which is static with respect to the acquisition (Andersson, Skare, Ashburner, 2003, Jezzard, Balaban, 1995). Given the dynamic nature of the distortion field in foetal MRI, as well as other abdominal applications, a dynamic estimation of the field map is required to allow dynamic distortion correction.

**Fig. 5:**
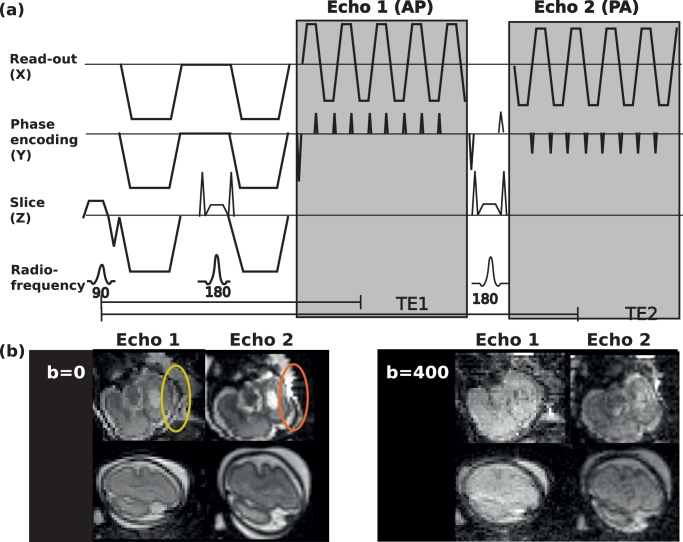
(a) Sequence diagram (simplified) of the double spin-echo sequence illustrating the gradient objects on Read-out (X), phase encoding (Z) and slice (Z) axis as well as radio-frequency pulses. (b) Acquired echoes with opposed phase encoding and thus equal-opposite distortions. (For interpretation of the references to colour in this figure legend, the reader is referred to the web version of this article.)

The proposed modified EPI sequence features a double spin echo, as previously proposed by Gallichan et al. (2010), with the second echo obtained with opposed phase encoding direction (see Fig. 5). While differing in echo time and thus contrast and signal, the two echoes have matched read-out bandwidth but opposite susceptibility induced shift effects/distortions. Their temporal proximity of  < 100ms and the need for a coherent signal pathway throughout the sequence to obtain signals, ensures that the two images produced per slice can be relied upon to have closely matched (nominally identical) motion states. Susceptibility induced stretching in the first echo (Fig. 5 yellow) corresponds to signal pile-up in the second echo (red).

### 2.5 Dynamic distortion correction

**Fig. 6:**
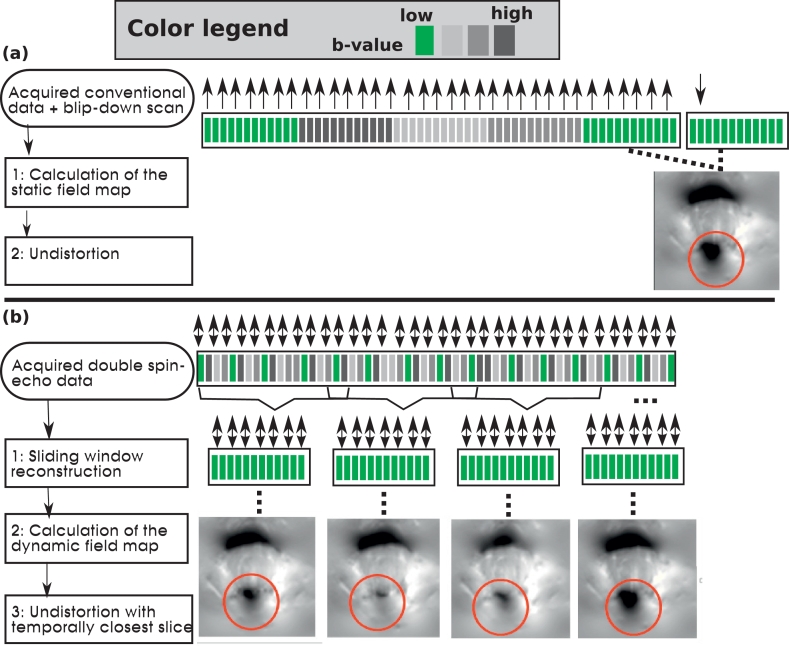
The postprocessing step required to correct for geometric distortions is depicted. In (a), a conventional technique is depicted: The dMRI scan acquired with one phase encoding direction (here AP) is complemented with a subsequent single b=0 volume with opposed phase encoding direction (here PA). Correction consists of (1) the calculation go a static field map and subsequent (2) correction of all volumes using this map. In (b) the proposed approach based on double-spin echo Superblock & Interleaving data is depicted: (1)Sliding window reconstruction extracts b=0 volumes throughout the dataset. These are used to calculate (2) dynamic field maps for all time steps which are finally (3) used to correct every slice with the temporally closest map.

Fig. 6a depicts conventional static distortion correction based on a B0 field estimate determined using a phase-encoding reversed b=0-volume applied to single phase-encoding dMRI acquisition as described above. The combination of a second echo and the superblock & interleaving diffusion sampling allows for a more dynamic approach as illustrated in Fig. 6b. The acquired superblock & interleaving double spin-echo data point pairs represent individual samples, differing by geometric location and diffusion weighting. The data is first re-ordered to assemble low *b*-value volumes using a sliding window approach (step 1, Fig. 6b). Each temporal volume *T_v_* gathers together the temporally closest low-*b* slices. The window size equals the superblock length, *L*, and the maximal temporal distance to measured distortion correction data thus equals 2TR for the case of L=5 illustrated. This data is used (step 2 Fig. 6b) to calculate field maps (in Hz) for every time point using FSL topup (Andersson et al., 2003).

In step 3 (Fig. 6b), the temporally closest field map is chosen to correct each slice for distortions. This operation is performed in scanner coordinates and the field maps are converted into displacements in mm taking the bandwidth of the sequence and the EPI factor into account.

### 2.6 Postprocessing—motion correction

**Fig. 7:**
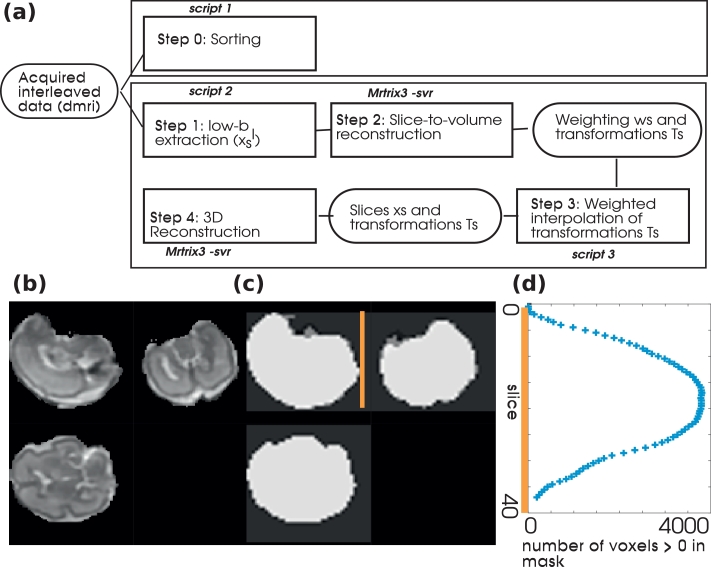
(a) Schema of the proposed postprocessing of the Superblock & Interleaving data. The first row, consisting only of step **sorting** sorts the data to a conventional volume-view data set. In the second row, a four-step pipeline including motion correction is depicted. In (b)–(d), the weighting approach is illustrated. (b) Depictes the b0 result as obtained after super-resolution reconstruction, (c) the derived brain mask and (d) the obtained number of voxels within the mask as input for the calculation of the weights.

In Step 1 (Fig. 7) all the low-*b* slices are combined as input to a SVR alignment. A brain mask is obtained by thresholding of the voxel intensities, followed by largest connected component analysis and median filtering - all implemented using MRtrix3 image processing commands (Tournier et al., 2012). A manual refinement step is performed, which is required especially in cases of anterior placentas.

Then, SVR implemented in MRtrix3 (Step 2, Fig. 7) is employed (Tournier et al., 2012). The process intersperses a registration step to progressively refine the position estimate of each slice in anatomical space, with a 3-D reconstruction step that uses all newly aligned data to generate a 3D volume that can then be used as a registration target for the next iteration. While this is not the focus of this study, the used reconstruction algorithm allows for super-resolution reconstruction to increase the target resolution. To aid convergence, the temporally closest b=0 slices are combined to b=0 volumes and registered in a first volume-to-volume registration until finally every individual slice is registered. This allows the position of each slice to be refined while accounting for temporal proximity. The outcome of this processing step are a motion-corrected low-*b* volume, transformation parameters for each individual low-*b* slice, and a weight assigned to each slice. This weight depends on the number of voxel within the brain mask for every slice. The fewer voxel within the mask, the less stable the registration and especially the less robust regarding rotation parameters. Therefore, the weights are obtained as the number of voxel within the brain mask divided by the number of total voxel per slice. (See Fig. 7b–d.) Any slices with less than 1% of voxels within the brain mask are given a weight ”0” and thus effectively marked as outliers.

In Step 3 (Fig. 7), all high-*b* slices are individually assigned a positional transformation obtained by weighted interpolation of the transformations for the two closest low-*b* slices in time. Specifically, rigid transformation matrices *R*_0_ and *R*_1_ are interpolated linearly at time 0 < *t* < 1 as:

(2) $$R\left(t\right)=\exp(t\log\left(R_{1}\right)+\left(1-t\right)\log\left(R_{0}\right)),$$

where *exp* and *log* are matrix exponential and logarithm. This interpolates a smooth trajectory and preserved the rigid transformation.

Finally, in step 4 the interpolated motion estimates are input to a 4-D reconstruction of the full DWI data in the spherical harmonics (SH) basis for every shell, that also accounts for necessary gradient reorientation due to motion. This 4-D reconstruction directly extends the least-squares conjugate gradient method described above to estimate, for each voxel, a vector of SH coefficients from the scattered slice data at given rigid motion parameters (Kuklisova-Murgasova et al., 2017).

## 3 Experiments

Experiments to explore and validate the proposed methods were conducted on a 3T Philips Achieva scanner running release 3.2.2 software and a 1.5T Philips Ingenia scanner running release 5.17 software. Software modifications were implemented to achieve the required enhancements to standard acquisition capabilities on both systems. All subjects gave written informed consent according to local ethics committee approved protocols.

### 3.1 Equivalence experiment

All experiments described below were performed on the clinical Achieva scanner.

To validate the sequence and test whether moving from a conventional diffusion encoding by complete volume to slice-wise diffusion encoding causes performance issues, both phantom and adult experiments have been performed: A sphere phantom was shimmed using image based shimming and scanned in the 32-channel adult head coil both using the conventional volume and the superblock acquisition. Next, a healthy compliant adult was scanned with the 32-channel adult head coil. Volume shim as well as fat saturation was applied.

Further fixed imaging parameters for both included resolution 2.2 mm isotropic, TR=5500 ms, TE=80 ms, SENSE=2, partial Fourier = 0.8, FOV =200 × 200 mm (220 × 200 for the adult experiment).

The presented superblock & interleaved scheme was employed on both phantom and adult. The diffusion encoding scheme was thereby matched to the scheme which was sampled for later foetal usage: 50 directions on 3 shells (b=0, b=400 and $b=1000\frac{s}{{\text{mm}}^{2}}$). The acquisition time was TA=4:40 min.

In scan 1, the conventional scan was performed sampling the encodings per volume: b=0, b=400, b=1000, b=1000, b=1000, in scan 2 the proposed superblock scheme with L=5 was used. Scan 2 was sorted to conventional volumes only, without any motion correction performed and then apparent diffusion coefficient (ADC) and fractional anisotropy maps were calculated using the diffusion MRI processing package MRtrix3 [Website www.mrtrix.org , Tournier et al. (2012)] for both conventional and superblock scan. The obtained quantities were compared on a voxel-by-voxel basis.

Finally, to illustrate the flexibility of the proposed method, 2 additional scans were performed on the adult. First, a *b*-value sweep was performed to illustrate the flexible slice level allocation of *b*-values. Therefore *b*-values between 0 and 2000, sampled in steps of 100 were acquired within the same volume, in addition one volume with b=0 and one volume with b=2000 was acquired within the same scan. Additional parameters included $N_{s}=40$ and acquisition time 21 s. Next, a multiband accelerated scan (multiband factor 2) was performed with the above presented superblock & interleaving acquisition. The TR was kept constant for illustration purposed resulting in the same acquisition time TE=4:40 min.

### 3.2 Simulations

**Fig. 8:**
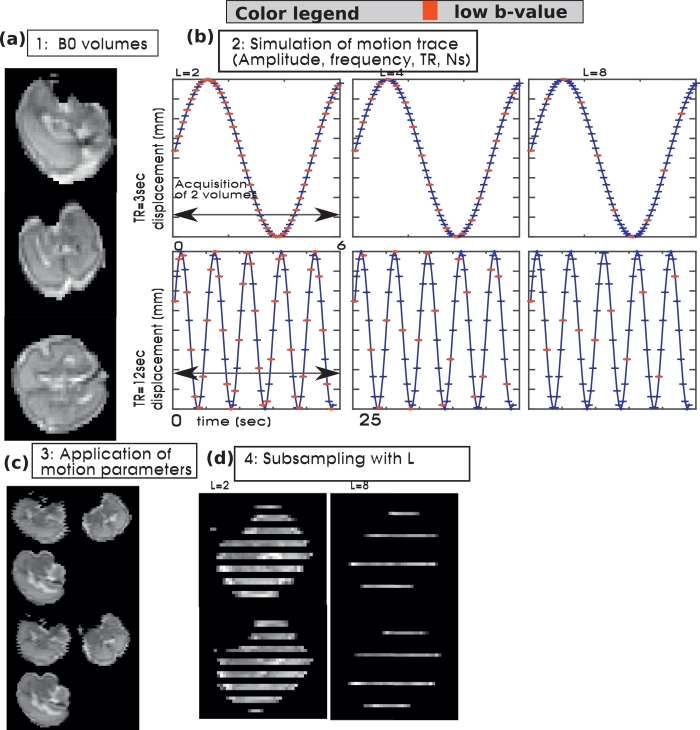
Results from the simulated breathing experiment are depicted. In (a), the obtained motion free volume is shown in sagittal, coronal and transverse plane. In (b), the simulated motion patterns for different repetition times (TR=3sec first row and TR=12sec second row) and different interleave patterns (L=2 first column, L=4 second column and L=8 in the third column) are shown. All capture the time frame of two subsequent volumes, the red dots illustrate the temporal acquisition order of the low-b data. (For interpretation of the references to colour in this figure legend, the reader is referred to the web version of this article.)

Tests to explore motion correction by interpolation of transformations from low-b slices regularly spaced in time were performed by simulating a motion affected dataset as follows: A motion-free b=0 volume was obtained as a result from slice-to volume reconstruction using foetal data acquired as described below. This volume was replicated $N_{d}=50$ times (step 1, Fig. 8) to make an image time series. Breathing motion with a breathing cycle length of 5sec was simulated as a sine-wave with a 5sec period and amplitude 4mm. To illustrate the effect of the number of slices and coverage, two versions, one with $N_{s}=30$—containing only the foetal brain and one with $N_{s}=40$—containing several slices above and beyond the brain were simulated with varying *TR*=3,6,12 and 15 s (step 2, Fig. 8). The resulting curve was sampled at $\Delta_{t}=TR/N_{s}$sec. In step 3, the simulated breathing curves were applied as *y*-translation following the given temporal order. Finally, all possible superblock lengths L=[2,3,5,6] for $N_{s}=30$ and L=[2,4,5,8] for $N_{s}=40$ were applied in step 4. This results in 32 different simulations.

The resulting motion-corrupted data is processed with the pipeline as specified in Fig. 6a and the mean deviation between input and output translation parameters over the entire $N_{d}=50$ volumes is assessed.

### 3.3 Foetal diffusion data

8 pregnant volunteers (gestational age 26–34 weeks) were studied as part of the developing Human Connectome Project (dHCP^1^) after informed consent was obtained (LO/1047). All women were imaged in a supine position (Hughes et al., 2016) using a 32-channel cardiac coil. The proposed double spin-echo Superblock & Interleaving diffusion sequence was used to acquire 3-shell HARDI data with a total of 49 directions (11 b=0, 8 b=400, 30 $b=1000\frac{s}{{\text{mm}}^{2}}$), isotropic resolution 2.2 mm^3^, 35–44 slices/volume, $N_{i}=$4–6, TR=11000–15000s, TE=107 ms for the first echo and 208 ms for the second echo, SENSE 2.0, using image-based shimming and SPIR fat suppression. The acquisition time varied slightly with varying slice number and TR between TA=9:09 min and TE=12:30 min.

## 4 Results

### 4.1 Equivalence experiment

**Fig. 9:**
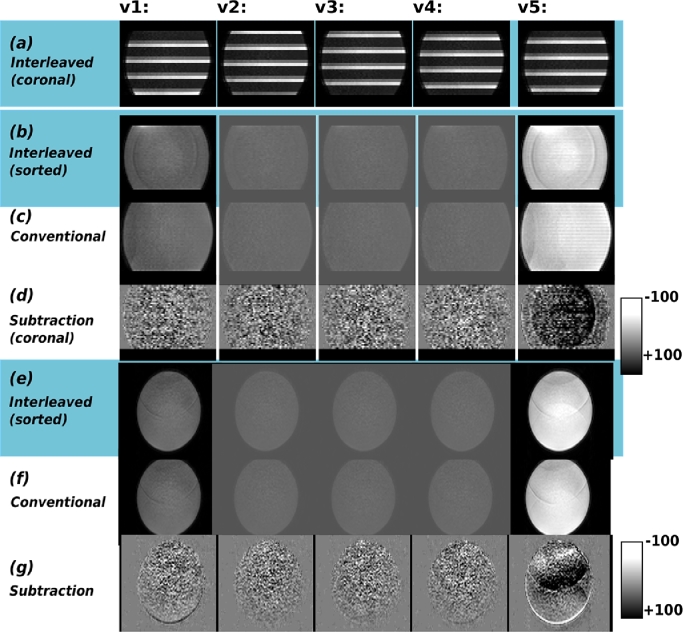
Results are presented from the phantom experiment. Thereby (a) illustrates the acquired data in coronal plane for the superblock scheme. (b) shows the data after sorting to conventional volumes in coronal view. In (c) the conventional data is displayed equally in coronal view. Sorted superblock and acquired conventional data at a mid-stack location in the axial plane is given in (e) and (f). Finally in (d and g) he difference between conventional and superblock is shown (scale 5% of the original data) in coronal (d) and axial (g) view.

**Fig. 10:**
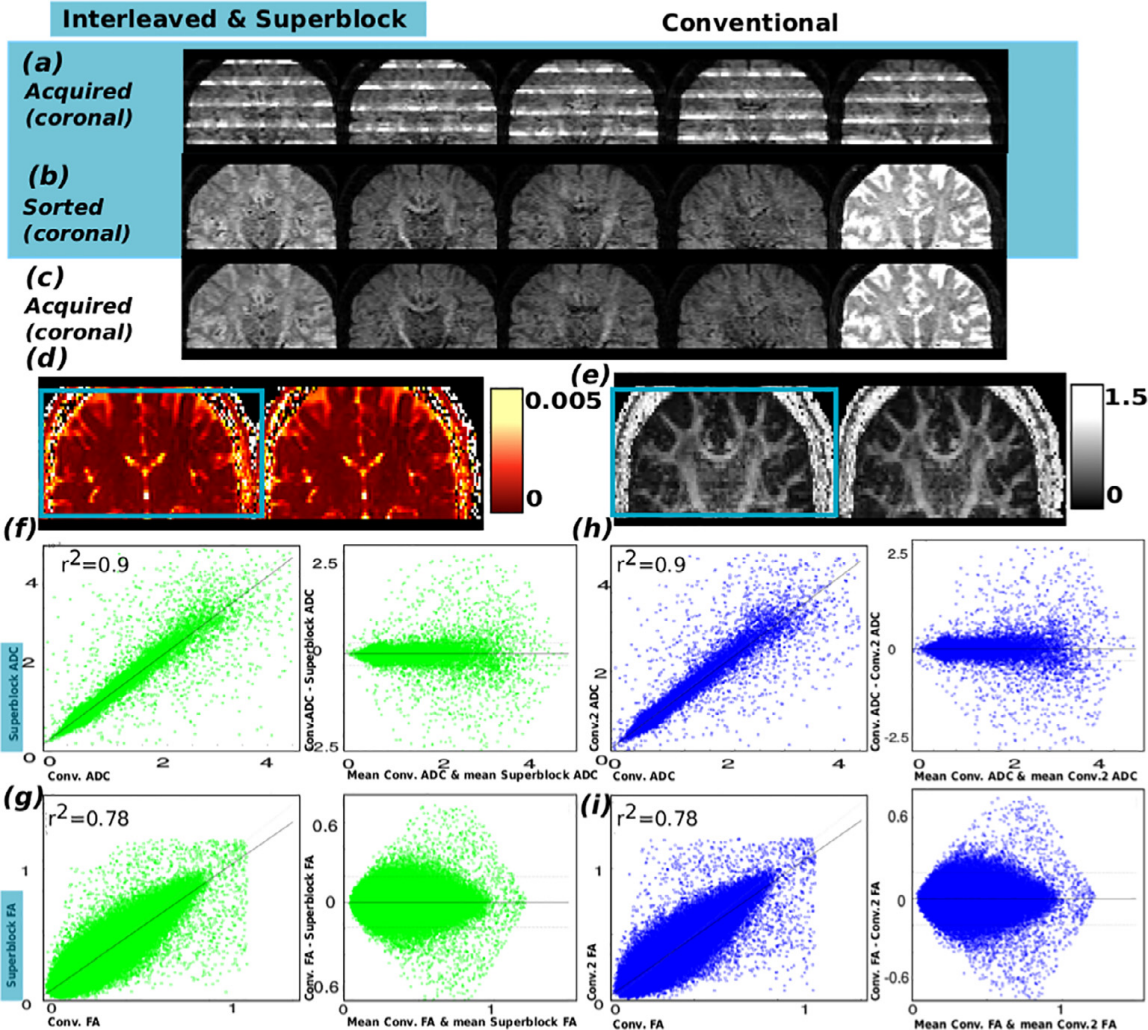
Results are presented from the healthy adult experiment. Thereby (a) illustrates the data in reformatted coronal plane for the interleaved acquisition. (b) shows the data after sorting to conventional volumes in coronal view. In (c) the conventional data is displayed equally in coronal view. Obtained ADC (d) and FA (e) maps are given for both acquisition types. Bland-Altmann plots for another subject are given in (f)–(i): Thereby the results from the conventional vs. interleaved experiments are given for ADC and FA in (f) and (g), Bland-Altman plots for the conventional vs. repeat conventional in (h) and (i) using all voxels within the brain mask.

The results from the phantom experiment for both scan 1 and 2 are given in Fig. 9. This includes for superblock data both the acquired in coronal (a) plane and sorted data in coronal (b) and axial plane (e). For conventional data the respective orientations are given in (c) and (f). Finally, the difference, displayed at 5% of the original signal intensity is given in (d) and (g). No systematic differences are observable between the two acquisitions. The results from the described adult experiments are given in Fig. 10. In (a) superblock acquired, (b) sorted and in (c) the conventional acquisition. Resulting ADC (d) and FA (e) maps are given for both acquisitions together with Bland-Altmann plots (f-i) of all the voxels in the brain mask. Thereby, the results from the interleaved vs. conventional test are given in (f) and (g), the results for conventional vs. conventional repeat in (h) and (i), both results in $r^{2}=0.90$ for ADC and $r^{2}=0.78$ for FA. The Bland-Altman plots confirm that there is no bias between the two methods (Table 1).

**Table 1:** Symbols and abbreviations.

$t=1\cdots N_{t}$	Slice index (within the entire acquisition)
$d=1\cdots N_{d}$	Diffusion encoding index
$v=1\cdots N_{v}$	Volume index
$s=1\cdots N_{s}$	Slice index (within one volume)
$l=1\cdots N_{l}$	Superblock index
$e=1\cdots N_{e}$	Echo index
*L*	Super block length

**Fig. 11:**
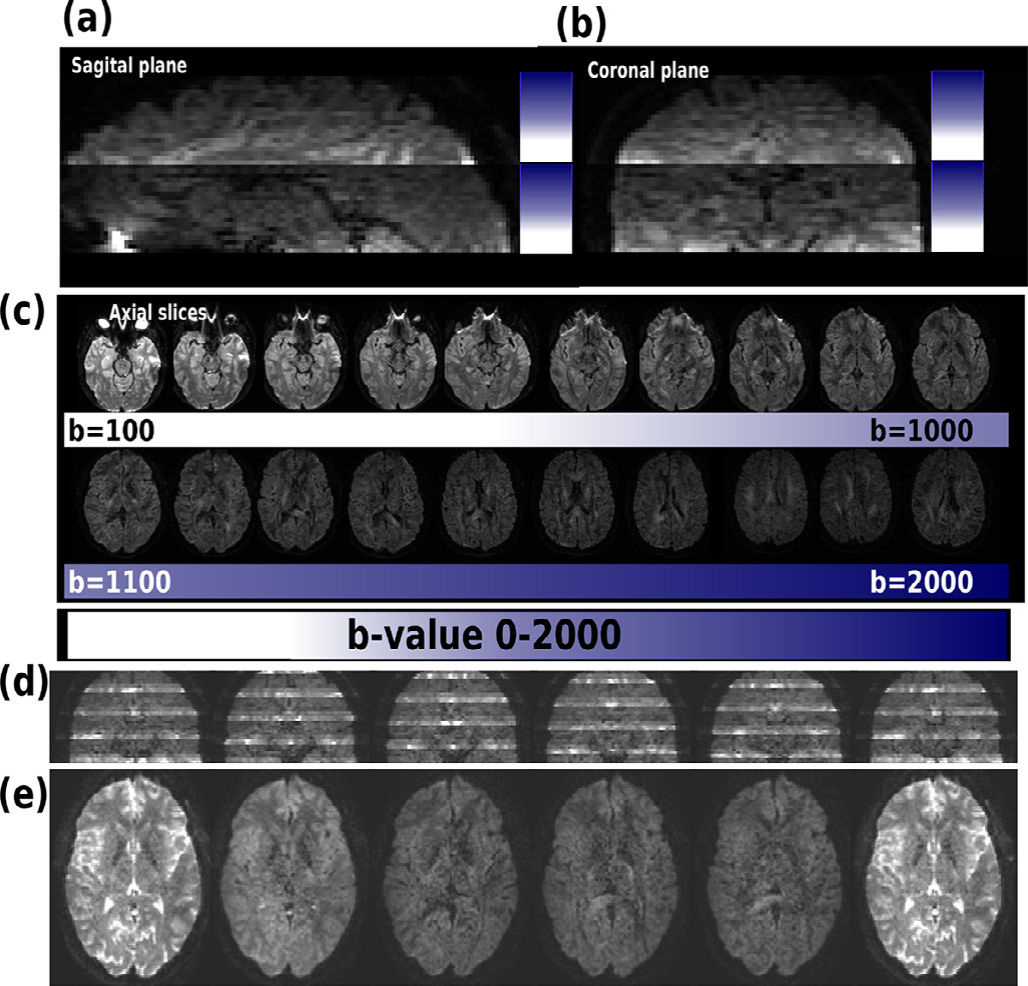
Results from the (a–c) b-value sweep experiment and the multiband acquisition (d–f) are shown on an adult brain. The sweep results are displayed reformatted in (a) sagittal and (b) coronal plane. (c) visualizes axial slices ranging from b=0 (left) to b=2000 (right). The reconstructed multiband data in coronal (d) and mid-stack axial orientation (e) is given.

Additional performed experiment results are given in Fig. 11. Thereby, in (a–c) reformatted coronal and sagittal planes from a b-value *sweep experiment* show the successful acquisition of 20 b-values between b=0 and b=2000 within the same volume in 20 s. As the number of slices was $N_{s}=40,$ every b-value was acquired twice - resulting in the two sweeps visible in the reformatted sagittal and coronal plane (a–b). The 20 separate b-values are visualized in spatially proximal slices in (c). Finally, Fig. 11d–e show results from the multiband experiment, illustrating the successful combination of the proposed sequence changes with multiband acceleration. Here, $N_{s}/L=2$ and the multiband factor was chosen as 2, therefore, the dataset is visually split in 4 blocks.

### 4.2 Simulations

**Fig. 12:**
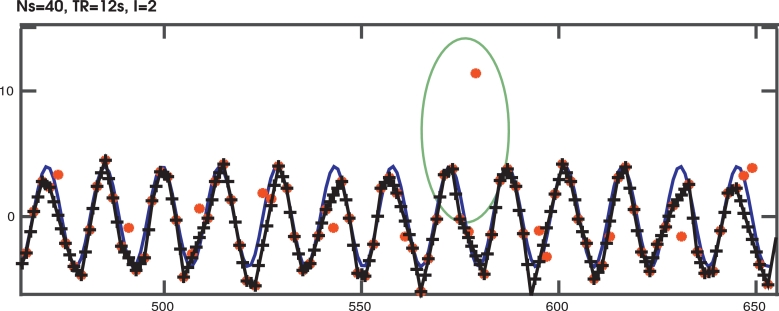
Resulting y-displacement curves overlaid over the sampled respiratory displacement curve are given exemplary for an acquisition with L=4. Thereby, the input displacement (ground truth) is given in blue, the transformation parameters obtained after registration of the low-*b* slices in red, and the interpolated transformation parameters for all slices in black. The green circle focuses on an outlier in the estimated transformations. It corresponds to a b=0 slice, located at the border of the spatial volume. The employed weighting strategy did not include these transformations in the interpolation routine. (For interpretation of the references to colour in this figure legend, the reader is referred to the web version of this article.)

The results for the simulations are given in Fig. 12 and Table 2. Thereby, the accuracy, given as the distance between sampled and obtained y-displacement was analysed.

**Table 2:** Simulation parameters and results. The number of b=0 samples per respiratory cycle - resulting from the sampling frequency assuming a cycle length of 5s - (pc), are given for $N_{s}=30$ slices for the L=2 case for repetition times TR=3,6,9,12. For all of the studied options, the mean error between simulated and obtained displacements are given in mm (shaded in grey).

TR		TR=3s	TR=6s	TR=9s	TR=12s
slice time		100 ms	200 ms	300 ms	400 ms
30 slices					
		25pc	13pc	8pc	6pc
Mean error [mm]		0.24	0.34	0.61	1.22

For the shown case of a TR of 12sec and L=2, the input *y*-displacement (blue in Fig. 12) is well recovered by the obtained low-*b* parameters (red points), subsequent interpolation led to the y-displacement parameters (black points and line). This setting includes 6 points per respiratory cycle (see Table 2).

The point within the green cycle was classified as an outlier according to the process described in the methods: The slice at this temporal location was at the edge of the volume (identified by  < 1% of voxels within the brain mask). Consequently, this parameter was excluded by the automatic weighting.

Quantitative results for all simulation settings are given in Table 2, illustrating that the method performs well for reasonable sampling density (  ≥ 5 low-*b* points per respiratory cycle).

The given mean values were obtained from both low-b and high-b values, but excluding the points corresponding to *z*-locations with  < 1% of the voxels within the brain mask.

### 4.3 Dynamic field mapping

**Fig. 13:**
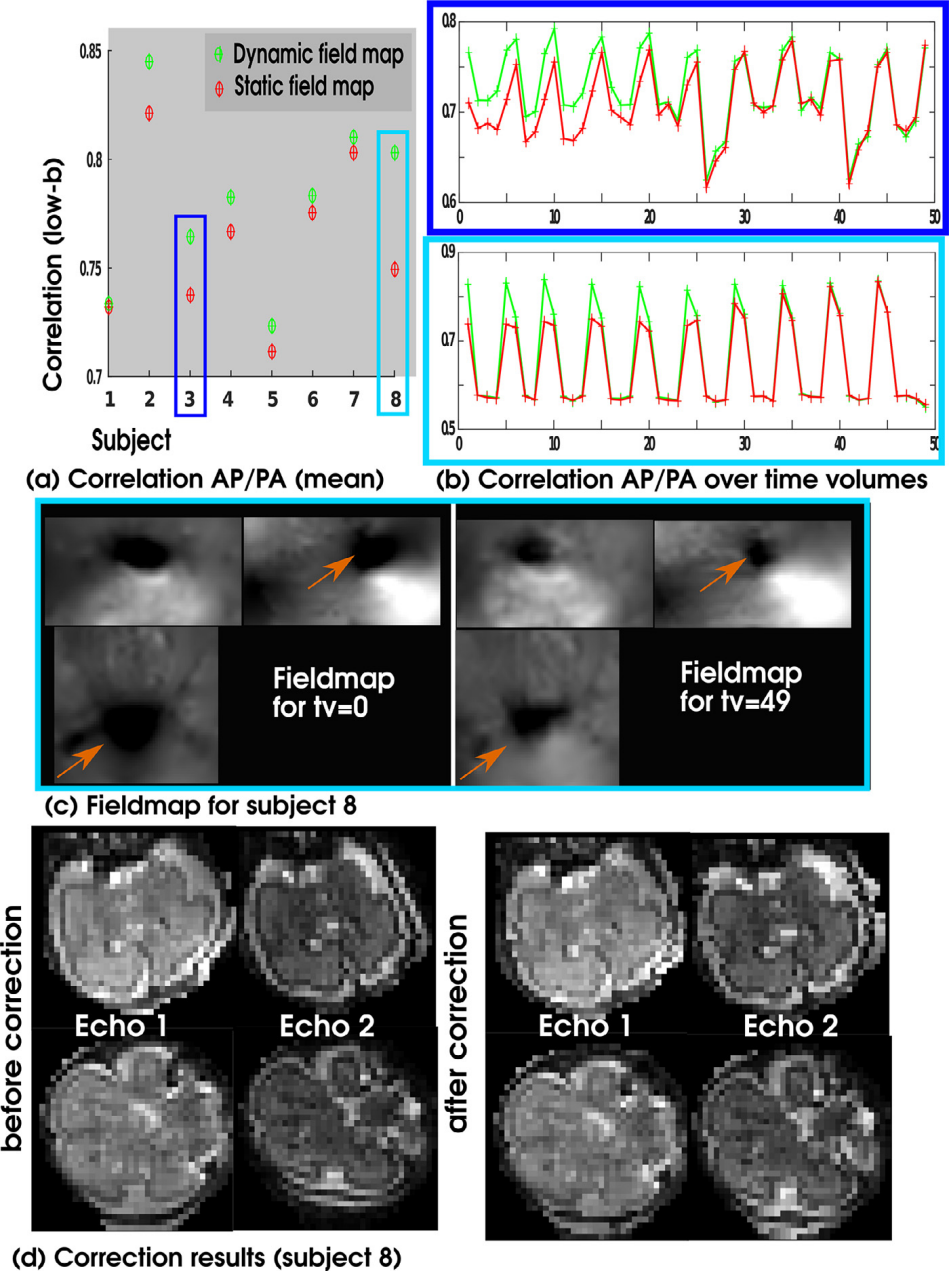
Results from distortion correction using the dynamic maps. (a) Mean over the correlations between AP/PA images for all low-*b* volumes are shown for the dynamic field map (green) vs. the static fieldmap acquired at the acquisition end (red). (b) The correlation for every diffusion weighting is shown for both corrections for subject 3 and 8. (c) Fieldmaps from the acquisition start and end and (d) correction results are shown for subject 8. (For interpretation of the references to colour in this figure legend, the reader is referred to the web version of this article.)

The dynamic calculation of a field map based on sparse but frequently acquired b=0 slices provides significant improvement in the presence of motion or varying B0-fields (e.g. as a result of intestinal gas bubbles). To assess the goodness of the distortion correction based on these maps, the data from both echoes was distortion corrected twice: (i) with the obtained dynamic field map using the described acquisition and processing steps, and (ii) using a static field map obtained from a conventional acquired double spin-echo pair at the end of the acquisition. The data from both phase encoding directions was then vectorized and their correlation coefficient per diffusion direction calculated. The time series of mean correlations for the low-*b* volumes is shown in Fig. 13a. The correlation per volume for static (red) and dynamic (green) distortion correction in (b) show that dynamic field mapping achieves consistently high correlations. The short term oscillations reflect intrinsic variation in correlation caused by the different SNR of low and higher *b*-value data. Static distortion correction improves towards the end of the series, which is when the static field map was acquired.

The upper panel in Fig. 13b illustrates a case with extensive foetal motion, illustrating improved correction for all volumes in the proposed approach. The lower panel in Fig. 13b illustrates a case where foetal motion is limited but the field map changes over time due to maternal bowel gas movement, as shown in Fig. 13c at the start and end of the sequence. Here, the proposed method significantly improved the consistency of the low-*b* volumes. Finally, the data from both echoes is shown before and after correction in (d), indicating high degree of geometrical consistency that is achieved between the echoes (a sign of precise distortion correction). The proposed correction framework was successful in all the subjects studied.

### 4.4 Derived quantitative dMRI information

The final dynamic distortion and motion corrected data is suitable for advanced dMRI analysis, including tractography and microstructural modelling. Here, we assess the overall quality of the data using conventional diffusion tensor imaging (DTI) and using a multi-shell spherical factorization (Christiaens et al., 2016) with two tissue components for brain tissue (SH order 4) and free water (isotropic). The overview of the motion parameters for all considered eight foetal datasets is given in Table 3. These were calculated as the root mean square of the forward difference of the motion parameters.

**Table 3:** Motion parameters for the foetal diffusion data.

ID	GA	*N_s_*	Motion
1	27+0	35	3.622
2	24+6	35	3.875
3	34+1	44	5.022
4	29+1	36	5.830
5	27+2	36	6.370
6	27+3	35	7.354
7	26+1	44	7.396
8	25+6	35	7.422

**Fig. 14:**
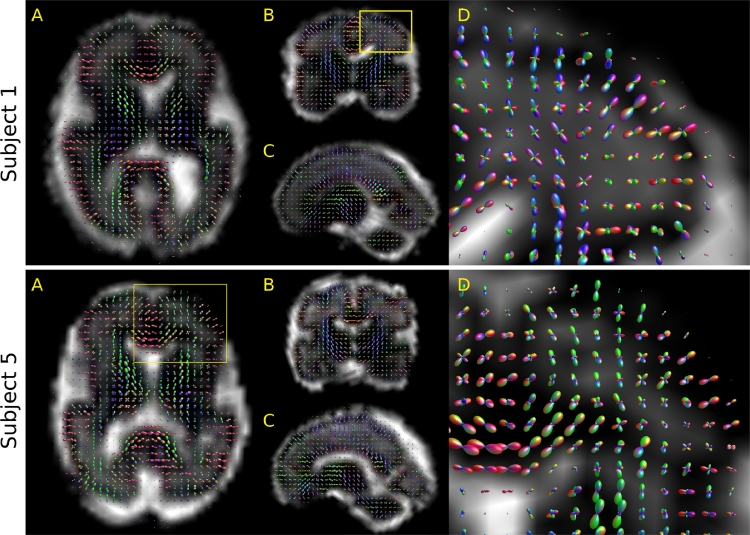
Orientation distribution functions (ODFs) overaid onto the estimated free water fraction in subjects 1 and 5. (A-C) Axial, coronal, and sagittal slices through the brain, rendered off-axis w.r.t. the acquisition. (D) Magnified ODFs from the yellow frame. (For interpretation of the references to colour in this figure legend, the reader is referred to the web version of this article.)

Fig. 14 shows tissue orientation distribution functions (ODFs) of subjects 1 and 5. These results show high anisotropy in the cortex and maturing white matter structures such as the splenium, as expected in early brain development. The ODFs are well aligned with developing white matter structures and with cell development perpendicular to the cortical surface. These results illustrate the practical applicability of our method in clinical assessment.

## 5 Discussion and conclusion

A novel flexible ssEPI diffusion sequence was presented, adopting a true *slice* view by allowing a fully independent choice of diffusion encoding per slice. Therefore, the *one volume – one encoding* paradigm was abandoned. One possibility to exploit this new flexibility was presented: the **superblock & interleave** approach. This approach optimizes the temporal sampling of low-*b* slices while ensuring that volumes are completed in case of early scan interruptions. Possible parameters within this approach were introduced and presented.

This acquisition was combined with a phase-encoding reversed second echo to allow dynamic motion correction. Finally, these novel acquisition elements were combined with a proposed slice-based processing pipeline. The approach allows dynamic distortion correction with a data derived field map generated every *N_i_* TRs, which for the examples shown means the closest distortion estimate is only 2TR, or 22–30 sec, distant. Motion correction estimates interpolate between low-*b* slices that are (*N_i_*/*N_s_*) ·  TR apart, which for the examples shown is around 1.6 s.

The results from the phantom and adult experiments show no signs of introduced artefacts or inconsistencies. The high correlation for the derived diffusion quantities ($r^{2}=0.95$ for ADC and $r^{2}=0.86$ for FA) shows close agreement between the novel slice-independent acquired data and conventional volume data.

In the foetal datasets studied so far the approach proved robust and effective, with clear evidence of both distortion and motion correction found in each subject. Simulations including a variety of parameter ranges illustrate the performance and the limits of the proposed method.

### 5.1 Extension in applications and parameter choices

The proposed changes to the sequence are independent of further sequence choices. They combine naturally with any choice of both diffusion encoding and read-out. This specifically includes any novel diffusion encoding such as *b*-tensor encoding (Szczepankiewicz et al., 2015), oscillating gradients or double refocused diffusion weighting as well as acceleration strategies such as parallel imaging (Griswold et al., 2002), multiband imaging (Setsompop et al., 2012) or multiplex imaging (Feinberg et al., 2010).

Furthermore, the proposed methods were demonstrated on foetal imaging but are by no means restricted to this application. Any diffusion MRI study suffering from motion artifacts and/or time varying susceptibility can benefit.

### 5.2 Extension towards more flexibility

The implemented sequence allows complete flexibility regarding diffusion encoding and geometric location on a slice-level. In the presented study, however, only the superblock & interleave scheme was presented. Further schemes varying the diffusion encoding not in an interleaved but more random or pseudo-random way can be thought of. Furthermore, variation in the slice location *z* in combination with a global inversion pulse at the beginning of each volume - generating in effect varying TR per slice can be employed in future studies to facilitate joint diffusion-relaxometry experiments.

### 5.3 Eddy currents

The strong, rapidly switching diffusion encoding gradients can give rise to additional off-resonance effects. The rapidly changing magnetic field induces eddy currents within conductors, inducing an additional magnetic field. The proposed flexible encoding changes the temporal order of the employed diffusion gradients, which could potentially complicate the expected eddy current behaviour. Our acquisitions have not shown any effect of increased eddy current artifacts, which builds confidence that the advantages shown for independent slice sensitisation are not associated with introduction of increased artefacts. Problems on different scanners are unlikely but can not be ruled out.

### 5.4 Additional constraints

Inclusion of sufficient low-*b* slices poses an additional constraint to the optimal sampling scheme. However, while the presented material only treated b=0 as low-*b* for simplification, this can be extended to higher-b values dispersed across lower shell(s) and thus add to the analysis. The choice of the threshold between low *b*-value (used for active distortion and motion correction) and high-*b* value (which are to be corrected) depends largely on the obtained SNR and the choice of registration and interpolation approaches employed. It may well be that optimal processing is achieved by using all data with appropriate weighting rather than the simplified approach of dividing into low and high b samples. This remains to be further explored.

Another constraint imposed by the superblock & interleave approach is a set relation between the number of slices *N_s_* and the chosen superblock length *L*, as *L* needs to be a proper divisor of *N_s_*. This condition is, however, easily achievable in most applications.

### 5.5 Scan time penalty

Specifically for the proposed combination with phase-encoding reversed second echo, an additional time penalty per slice of  ≥ 100*ms* is added. The real prolongation of scan time due to this depends, however, on the specific properties of the studied tissues. For the presented example, foetal imaging, the repetition time is largely set by the T1 of the foetal brain and resulting longer TR to achieve sufficient SNR. Therefore, the proposed acquisition combines synergistically with multiband acceleration (MB), since the theoretical TR reduction may not be achievable in foetal scanning due to decreased signal.

### 5.6 Future post processing advances

The presented paradigm-change from volume to slice-view provides more eloquent data. The proposed post-processing constitutes merely a first step to exploit these novel properties. A number of the proposed post-processing based algorithms as cited in the introduction might benefit from the additional information contained within the data. Further improvements, for example regarding outlier treatment or inclusion of higher shells in the motion parameter estimation would further increase the benefits of the proposed acquisition scheme.

To facilitate this, all tools for the described post-processing steps in Fig. 7 will be made available, either in the supplementary material (script 0,1,2) or within MRtrix3 together with exemplary data sets.

### 5.7 Thermal benefits

The proposed method allows to accelerate the acquisition time due to decreased gradient heating. However, for the data presented in this paper, we fixed the acquisition time of the proposed interleaved acquisition to the time of the conventional scan. The time required to acquire all slices for one volume (repetition time, TR) originates both from the time for playing the imaging gradient and the add-on time required for gradient heating. This add-on time is defined based on the worst thermal situation, calculated by assessing the thermal load over the entire sequence including all diffusion encodings. The highest load is typically achieved by repeating the most demanding diffusion gradients as required by the highest *b*-value.

The proposed adopted slice view also helps to mitigate this problem: The interleaving of high and low *b*-value slices limits sequential gradient demand and thus heating. Therefore, the required add-on time for gradient cooling is reduced. It hence allows more efficient scanning.

## 6 Conclusion and future work

The proposed free choice of diffusion encoding per slice rather then per volume breaks with the conventional one-volume-one-encoding approach and thus increases the flexibility of single-shot diffusion weighted EPI. Both simulations and experimental data acquisition were performed and combined with a matched data processing pipeline to demonstrate and test the proposed approach. The results presented illustrate the ability of this sequence to obtain matching quantitative MRI values to an equivalent conventional sequence. The increased flexibility to control the spatial and temporal distribution of low and high b slices offers advantages for motion and distortion correction, and this was illustrated using foetal diffusion data.

Further possibilities of the sequence were demonstrated with the acquisition of a single volume with 20 b-values and the successful combination with Multiband acceleration as illustrated in Fig. 11a–c (20 b-values) and Fig. 11d–e (multiband). The presented multiband data illustrates the compatibility, however a more systematic exploration of multiband acquisition with the proposed contributions is beyond the scope of this manuscript.

The benefits from the main contributions to the dMRI acquisition - interleaving of low-b slices and the second phase-reversed echo - have been illustrated and a first pipeline which can exploit the specific properties of such acquired data has been demonstrated on the post-processing side. However, this initial pipeline is a first attempt, significant improvements can be achieved by novel post-processing developments bespoke to the presented novel acquisition.

Future work will also include applications to other motion-challenging applications of diffusion MRI. Furthermore, additional benefits due to the reduction of thermal stress on the gradient system afforded by constantly changing the gradient demand from slice to slice will be exploited.

## Appendix A Supplementary materials

Supplementary Data S1 Supplementary Raw Research Data. This is open data under the CC BY license http://creativecommons.org/licenses/by/4.0/ Supplementary Data S1

## Footnotes

1 www.developinghumanconnectome.com
