# Supplementary figures and images for: Slice-level diffusion encoding for motion and distortion correction
Source: Med Image Anal. 2018 Aug;48:214–29. doi: 10.1016/j.media.2018.06.008 (PMC6191883; doi:10.1016/j.media.2018.06.008)

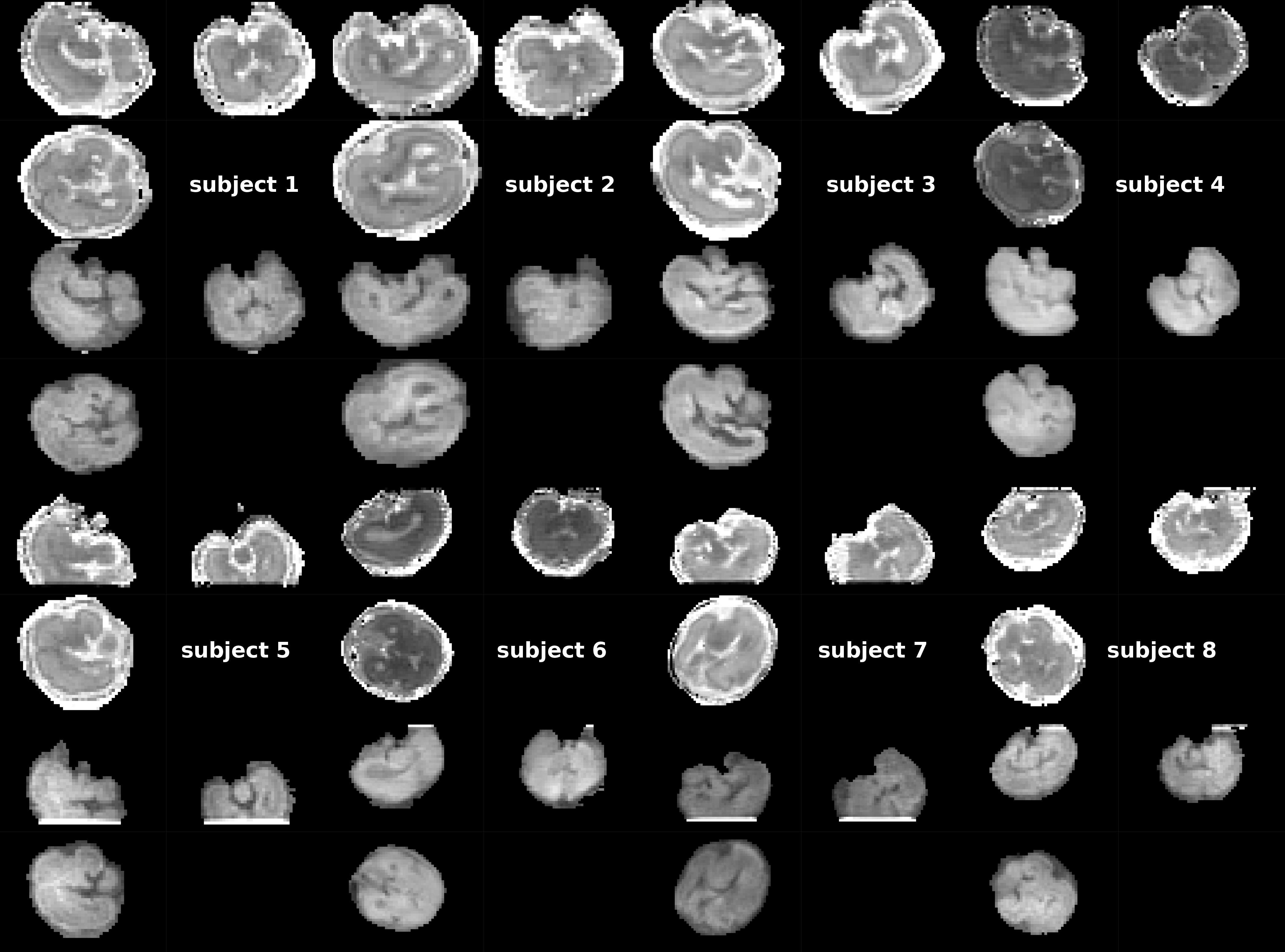

Supplement: Supplementary Data S1 — Supplementary Raw Research Data. This is open data under the CC BY license http://creativecommons.org/licenses/by/4.0/ [file mmc1.jpg]
